# The gene family that cheats Mendel

**DOI:** 10.7554/eLife.28567

**Published:** 2017-06-20

**Authors:** J Dylan Shropshire, Antonis Rokas

**Affiliations:** 1 Department of Biological Sciences, Vanderbilt University, Nashville, United States; 1 Department of Biological Sciences, Vanderbilt University, Nashville, United Statesantonis.rokas@vanderbilt.edu; 2 Department of Biomedical Informatics, Vanderbilt University, Nashville, United States

**Keywords:** meiotic drive, infertility, evolution, genetic conflict, segregation distortion, spore killer, *S. pombe*, Other

## Abstract

Some alleles of the *wtf* gene family can increase their chances of spreading by using poisons to kill other alleles, and antidotes to save themselves.

**Related research article** Hu W, Jiang Z-D, Sou F, Zheng J-X, He W-Z, Du L-L. 2017. A large gene family in fission yeast encodes spore killers that subvert Mendel’s law. *eLife* **6**:e26057. doi: 10.7554/eLife.26057

**Related research article** Nuckolls NL, Núñez MAB, Eickbush MT, Young JM, Lange JJ, Yu JS, Smith GR, Jaspersen SL, Malik HS, Zanders SE. 2017. *wtf* genes are prolific dual poison-antidote meiotic drivers. *eLife* **6**:e26033. doi: 10.7554/eLife.26033

Many of the traits an individual has, from eye color to the risk of having certain diseases, are passed from parents to their children via their genes. In diploid organisms, such as humans, most cells contain two copies – or alleles – of every gene. The exceptions to this rule are gametes (that is, sperm and egg cells), which contain just one allele. According to Mendel’s famous law of segregation, half of the gametes will carry one allele for a given gene, and the other half will carry the other allele. Thus, both of the mother’s alleles have an equal chance of being passed on to her children, and likewise for the father’s alleles.

However, some alleles defy Mendel’s law and can increase their chances of being transmitted to the next generation by killing gametes that do not share the same alleles (Burt and Trivers, 2006). Genes harboring alleles that behave in this way have been identified in plants, fungi and animals – including humans – and are called by various names, including selfish drivers, gamete killers and spore killers.

There are many types of selfish drivers and much remains unclear about how they work, though they can generally be distinguished by the way they destroy other cells. In the ‘poison-antidote’ model, the selfish driver produces a poison that destroys all gametes unless an antidote is there to protect them from the effects of the poison (Figure 1A and B). In the ‘killer-target’ model, the selfish driver produces a poison that kills gametes that carry a specific target (Figure 1C).

**Figure 1.**
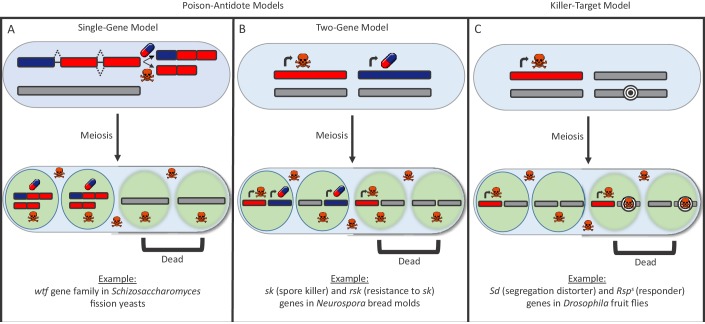
The poison-antidote and killer-target models of selfish drivers. In both models, a particular allele has an increased chance of being passed on to the next generation because it produces a toxin to kill gametes that do not carry it. (**A, B**) In the poison-antidote model, cells produce a toxin (shown as skull-and-crossbones) that can be neutralized by an antidote (shown as a pill); the alleles that do not code for either are shown in gray. In the single-gene model (**A**) the same gene codes for both the poison and the antidote through alternative transcription. Nuckolls et al. show that the gene *wtf4* is a selfish driver in *Schizosaccharomyces* yeasts. Hu et al. show that two other genes in the *wtf* family (*cw9* and *cw27*) are also selfish drivers. In the two-gene model (**B**) different genes produce the poison and antidote, as in the fungus *Neurospora* (Hammond et al., 2012). (**C**) In the killer-target model, the toxin only destroys cells that contain alleles with a specific target marker (shown here by concentric black circles). This is the case in *Drosophila,* where the segregation distortion (*Sd*) allele acts by killing gametes that contain a sensitive *Responder* (*Rsp^s^*) marker (Larracuente and Presgraves, 2012).

Aiming to understand how selfish drivers have evolved and work, two research groups – one led by Sarah Zanders and Harmit Malik, the other by Li-Lin Du – turned to two species of fission yeast, *Schizosaccharomyces kambucha* and *S. pombe.* These two species are genetically nearly identical, and some researchers do not even consider them as separate species (Rhind et al., 2011), but hybrids between the two are often sterile. In previous studies, Zanders and co-workers discovered that *S. kambucha* has at least three selfish drivers that cause infertility in the hybrids (Zanders et al., 2014).

To unravel the genetic identity of these selfish drivers in yeast, Zanders, Malik and co-workers at the Stowers Institute for Medical Research, the Fred Hutchinson Cancer Research Center and the University of Kansas Medical Center – including Nicole Nuckolls and Maria Angelica Bravo Núñez as joint first authors – isolated a region on a chromosome that caused selfish drive (Nuckolls et al., 2017). Within this region, they found a selfish-driver gene called *wtf4* – a member of a large and cheekily-named gene family – which creates both a poison and an antidote.

To explore the underlying mechanisms in more detail, Nuckolls et al. created fluorescent versions of the poison and the antidote and mapped their location inside and around the gametes. These elegant experiments showed that *wtf4*’s poison can leave their originating cells and cross into surrounding cells while the antidote remains trapped inside the cells that produce it.

In an independent study, Du and co-workers at the National Institute of Biological Sciences in Beijing – including Wen Hu as first author – identified two other genes from the *wtf* gene family, named *cw9* and *cw27*, as selfish drivers that also employ the poison-antidote model in crosses between different strains of *S. pombe* (Hu et al., 2017). They found that mutant diploid strains missing both copies of either *cw9* or *cw27* survived more than strains missing only one copy of the gene, indicating that both genes are selfish drivers. When they created diploid mutants missing a copy of *cw9* and *cw27*, the yeast strains survived even less compared to strains missing a copy of only one of the two genes. This suggests that the two genes do not rescue each other and that they act independently to drive survival.

By identifying several genes within the same family that can kill cells that are different, and by exploring their mode of action, the work of these two groups enriches our understanding of the genes that break Mendel’s acclaimed genetic law. Future work in this area will help us to understand the impact of selfish elements on genetic diversity and may lead to a deeper understanding of how these mechanisms affect conditions such as infertility in species as diverse as plants, fungi and animals – including humans.

## References

[bib1] Burt A, Trivers R (2006). Genes in Conflict: The Biology of Selfish Genetic Elements.

[bib2] Hammond TM, Rehard DG, Xiao H, Shiu PK (2012). Molecular dissection of *Neurospora* spore killer meiotic drive elements. PNAS.

[bib3] Hu W, Jiang Z-D, Sou F, Zheng J-X, He W-Z, L-L D (2017). A large gene family in fission yeast encodes spore killers that subvert Mendel’s law. eLife.

[bib4] Larracuente AM, Presgraves DC (2012). The selfish segregation distorter gene complex of *Drosophila melanogaster*. Genetics.

[bib5] Nuckolls NL, Núñez MAB, Eickbush MT, Young JM, Lange JJ, Yu JS, Smith GR, Jaspersen SL, Malik HS, Zanders SE (2017). *wtf* genes are prolific dual poison-antidote meiotic drivers. eLife.

[bib6] Rhind N, Chen Z, Yassour M, Thompson DA, Haas BJ, Habib N, Wapinski I, Roy S, Lin MF, Heiman DI, Young SK, Furuya K, Guo Y, Pidoux A, Chen HM, Robbertse B, Goldberg JM, Aoki K, Bayne EH, Berlin AM, Desjardins CA, Dobbs E, Dukaj L, Fan L, FitzGerald MG, French C, Gujja S, Hansen K, Keifenheim D, Levin JZ, Mosher RA, Müller CA, Pfiffner J, Priest M, Russ C, Smialowska A, Swoboda P, Sykes SM, Vaughn M, Vengrova S, Yoder R, Zeng Q, Allshire R, Baulcombe D, Birren BW, Brown W, Ekwall K, Kellis M, Leatherwood J, Levin H, Margalit H, Martienssen R, Nieduszynski CA, Spatafora JW, Friedman N, Dalgaard JZ, Baumann P, Niki H, Regev A, Nusbaum C (2011). Comparative functional genomics of the fission yeasts. Science.

[bib7] Zanders SE, Eickbush MT, Yu JS, Kang JW, Fowler KR, Smith GR, Malik HS (2014). Genome rearrangements and pervasive meiotic drive cause hybrid infertility in fission yeast. eLife.

