# Correction: Heredity: The gene family that cheats Mendel

**DOI:** 10.7554/eLife.31295

**Published:** 2017-08-22

**Authors:** J Dylan Shropshire, Antonis Rokas

Shropshire JD, Rokas A. 2017. Heredity: The gene family that cheats Mendel. *eLife* **6**:e28567. doi: 10.7554/eLife.28567.

Published 20, June 2017

The gene names in the example of the two-gene model provided on panel B of Figure 1 were incorrectly spelled and abbreviated. We have corrected this error by replacing the sentence "Example: *sp* (spore killing) and *rsp* (resistant to *sp*) genes in *Neurospora* bread molds" with the sentence: "Example: *sk* (spore killer) and *rsk* (resistant to *sk*) genes in *Neurospora* bread molds" on panel B of Figure 1. Please note that this correction does not affect the results and conclusions of the original paper.

The corrected Figure 1 is shown here:

**Figure:**
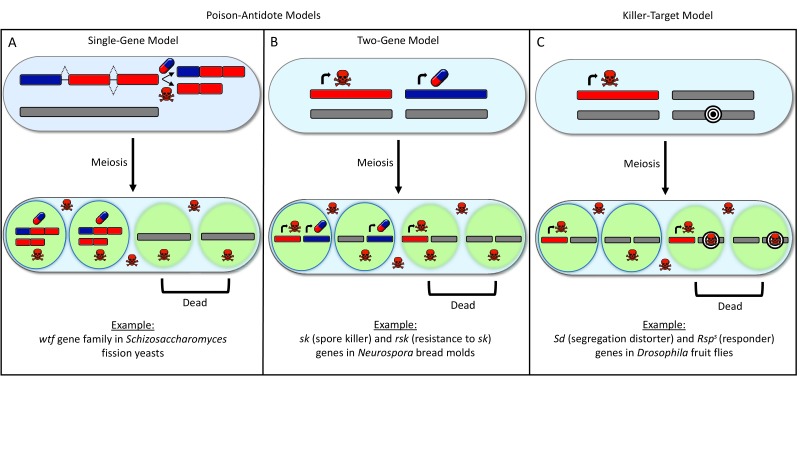


The originally published Figure 1 is also shown for reference:

**Figure:**
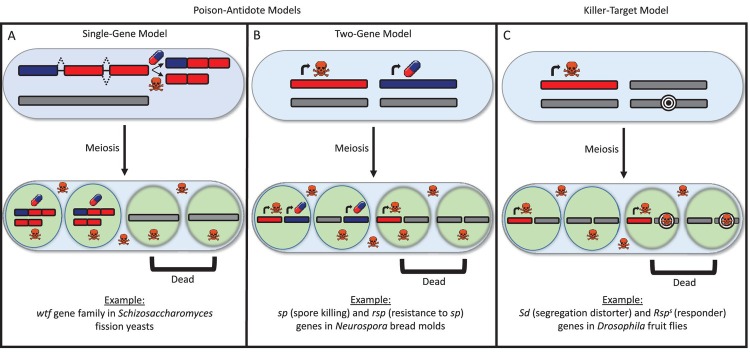


The article has been corrected accordingly.

